# Revisiting the need for intellectual and moral virtues in making ethical decisions in Healthcare

**DOI:** 10.12669/pjms.40.11.10602

**Published:** 2024-12

**Authors:** Noor-i-Kiran Naeem

**Affiliations:** 1 Dr. Noor-i-Kiran Naeem, FCPS, MSc. MEd, PhD Medical Education. Assistant Professor, Medical Education, ABWA Medical College, Faisalabad, Pakistan. Email: noorikiran@yahoo.com

**Keywords:** Ethics, Medical, Moral Development, Virtues, Decision Making

## Abstract

Patient-physician interaction is an essential factor influencing the patient’s health management decisions. This communication allows the patients to develop trust in the treating physicians and facilitates them in deciding what’s best for themselves. The physicians, on the other hand, may interact with the patients based on three moral philosophies i.e. as a part of their duty (deontology), as a part of anticipating consequences (consequentialism) and as a part of being a virtuous doctor (virtue ethics). In this communication, we aim to debate the need and utility of having moral virtues for ethical decision-making. We will also evaluate the role of intellectual virtues in patient-doctor interaction for decisions taken on clinical grounds.

## INTRODUCTION

**Fig.1:**
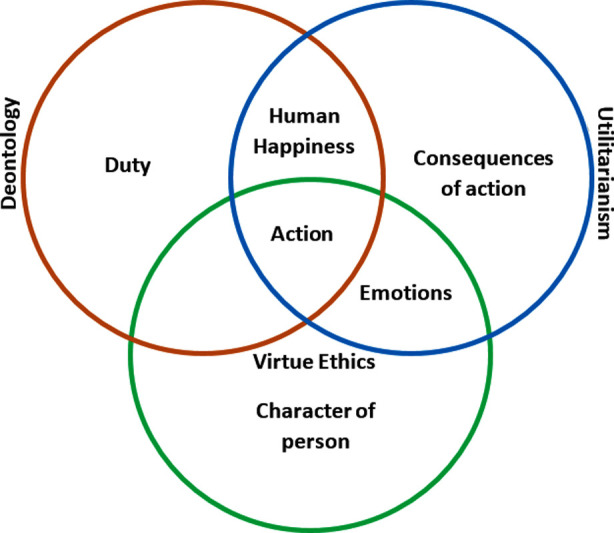
The Three Moral Theories.

Ethical theories can be divided into theories of conduct and theories of character, and virtue ethics focuses on the latter. Virtue ethics focuses on the betterment of agent of the action and not just the rule or action itself.^1^ Virtue ethics serves the purpose to establish an ethical community by developing good character among its people as shown by virtuous acts. (Fig.1)

Plato described virtue ethics as “*excellence of the soul containing reason, passion and spirit*”.^2^ His ideas were taken by Aristotle’s virtue ethics theory which explained that the life’s work and struggle done to achieve sense of happiness. This happiness, he defined, was achieved whenever there was a balance and harmony between reason and passion. He further introduced *Eudaimonia (flourishing/happiness)* as the final state of a human being, which is a rational, logical as well as purposeful self-realisation of intellectual and moral virtues.^3^ One can achieve happiness only if one fulfills the “*function appropriate to a human being which is to act in accordance with reason with virtuous intellectual and moral actions*”.^4^ Aristotle’s virtue ethics described the virtues as further being moral (character/disposition) and intellectual virtues.

### Intellectual virtues:

The intellectual virtues are the virtues or good habits of excellence of the mind that help the person decide and choose on grounds of his wisdom. These virtues can be taught over time via instruction and hence can be developed with external motivations. Much of the virtues include habits needed for getting started in some tasks, executing that task well and handling any challenges that may come in between. These may include prudence, art, wisdom or intelligence, and a practical knowledge of how to live.^4^

With the daily processes of decision making, they come into account when physician reflects upon the pros and cons of any decision about the patient (reflective practitioner) Among intellectual virtues that physician can acquire for decision making, is *phronesis* or *practical wisdom*, curiosity, intellectual humility, intellectual autonomy, attentiveness, intellectual carefulness, intellectual thoroughness, open-mindedness, intellectual courage, and intellectual perseverance.^5^

### Moral Virtues:

Moral virtues, on the other hand, are the good habits for achieving excellence of the soul. These are formed through process of habituation and hence require consistent practice of doing right actions for the right reasons. Moral virtues cannot be taught via instruction but acquired through practice. Some of the moral virtues include honesty, tempered ambition, wittiness, friendliness, generosity, civility, modesty and sincerity prudence, justice, fortitude, and temperance.^4^ Furthermore, these virtues can be categorized on the basis of lack or excess displayed by a person in course of action (which then become vices). While managing patients, it is important that optimum amount of moral virtue is displayed. For example, neither lying nor “truth dumping” are acceptable ways of promoting the virtue of honesty with patients.

### Importance of virtue ethics in Health care:

Virtue ethics focuses on development of character and enabling the person to manage his daily life decisions and choices through logic as well as passion, the skill which needs to be acquired through practice. It attempts to answer a fundamental question in ethics: *What sort of person should I be?*

On the other hand, the concept of health care provider and the professional virtues attached to him are defined by the society where he serves. These virtues though remaining somewhat the same, all around the world, can be influenced by the societal and cultural factors like religion, societal rules about ethnic tolerance and gender bias. For every physician working, it becomes necessary for him to self-reflect and ask himself, “*What sort of a doctor should I be?”* This can help him define the virtues he should develop in himself to be known as a doctor with good character. Same question can be asked while he deals with patients and must take any decision in clinical care, “*What decision is to be taken for this case which will be according to my virtues and character?*”

**Fig.2:**
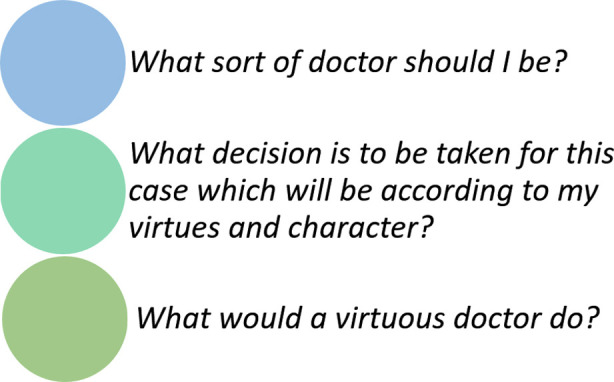
Questions for self-reflection for developing virtue ethics for health professionals.

The process of cultivating these virtues is ongoing and iterative as the doctor acquires training and experience. In nurturing these virtues, the doctor voluntarily and logically chooses to practice it, and allows space for the virtues to grow over time. All of this is not out of the necessity of being on duty, but in fact due to the innate motivation behind it to be virtuous. For a virtuous doctor, a life with happiness and fulfilment is the one where he takes decision driven by his virtues, and hence when faced by any ethical dilemma, he would ask himself,” *What would a virtuous doctor do?”* (Fig.2)

### Virtue ethics in ethical decision making:

Research have demonstrated three procedures involved in ethical decision making in virtue ethics.^4^

1. Use of different moral virtues over a long period of time to internalise the experience and act out of habit.
2. Differentiate between the excess and deficiency of each virtue and select an appropriate level of virtue after reflection- commonly known as the *golden mean*, thereby allowing the physician to use intellectual virtues.
3. Choosing the virtue that a virtuous doctor would use.

### Importance of Integrating Virtue Ethics into Medical Education:

There has been a significant push to integrate virtue ethics into medical education in recent years, particularly in the UK. The Institute of Medical Ethics’s model core curriculum (IME) highlights two primary objectives^6^:

1. **Creating ‘Virtuous Doctors’:** The first goal is to develop virtuous doctors who embody moral virtues such as honesty, integrity, and compassion. This initiative underscores the importance of nurturing character traits that align with ethical practice in healthcare. By emphasizing these virtues, medical education aims to prepare physicians with technical expertise and exemplary moral conduct. This approach recognizes that ethical practice is not solely about following rules or protocols but also about embodying the virtues that define good character.

2. **Providing a Skill Set for Analyzing and Resolving Ethical Problems:** The second objective is to equip medical professionals with the intellectual virtues necessary for analyzing and resolving complex ethical dilemmas. Practical wisdom (phronesis), intellectual humility, and critical thinking are crucial for navigating the nuanced and often ambiguous situations encountered in clinical practice. The focus on intellectual virtues ensures physicians can approach ethical challenges with a well-rounded perspective, integrating moral principles and practical considerations.

Acknowledging the importance of virtue ethics, the University of Health Sciences, Lahore, has recently introduced a spiral PERLs module (Professionalism, Ethics, Research and Leadership) along with a recent integrated module curriculum of undergraduate medical education.^7^ Spanning through all the MBBS years, the integration of virtue ethics into medical education can integrate the dual role of virtues in ethical decision-making. Moral virtues cultivate a character conducive to ethical practice, shaping how physicians interact with patients and make decisions. On the other hand, intellectual virtues provide the cognitive tools needed to analyse and address ethical issues effectively. This dual emphasis ensures that physicians are morally grounded and intellectually equipped to tackle the complexities of modern healthcare.

By embedding virtue ethics into the curriculum, medical education fosters a holistic approach to ethical practice. It encourages future physicians to reflect on their personal values and professional responsibilities, promoting a culture of integrity and excellence in healthcare. This comprehensive approach to ethics is crucial in preparing doctors who are skilled practitioners and principled individuals capable of making sound ethical decisions.

## CONCLUSION

Virtue ethics guides ethical decision-making by focusing on the central role of character, judgment and integrity deliberately developed over a lifetime. Both moral virtue and intellectual virtues are needed by the physician for ethical decision-making. It is difficult to perceive if a person is an intellectually very good reflective doctor but rude and dishonest with his patients. Hence, maintaining high levels of intellectual virtues will be of no use alone when a person does not exhibit high moral values and vice versa. Limited present work in literature presents a gap of knowledge which needs to be explored in future studies.
